# Validation of the Dermatologic Complexity Score for Dermatologic Triage

**DOI:** 10.3390/diagnostics15212765

**Published:** 2025-10-31

**Authors:** Neil K. Jairath, Joshua Mijares, Kanika Garg, Katie Beier, Vartan Pahalyants, Andjela Nemcevic, Melissa Laughter, Jessica Quinn, Swetha Maddipuddi, George M. Jeha, Sultan Qiblawi, Vignesh Ramachandran

**Affiliations:** 1 Department of Medicine, Indiana University, Indianapolis, IN 46290, USA; josmijar@iu.edu; 2 Philadelphia College of Osteopathic Medicine, Philadelphia, PA 19131, USA; kg3419@pcom.edu; 3 School of Medicine, University of Toledo, Toledo, OH 43614, USA; katie.beier@rockets.utoledo.edu; 4 Department of Dermatology, New York University, New York, NY 10010, USA; vartan.pahalyants@gmail.com (V.P.); andjela.nemcevic@gmail.com (A.N.); melissa.laughter22@gmail.com (M.L.); 5 Mount Sinai Department of Dermatology, New York, NY 10010, USA; jessquinn@mtsinai.org (J.Q.); swetha.maddipuddi@mtsinai.org (S.M.); 6 Summit Dermatology, Livonia, MI 48150, USA; gmjeha@gmail.com (G.M.J.); sqiblawi11@gmail.com (S.Q.); 7 Skin Institute of New York, New York, NY 10010, USA; vig.ramachandran@gmail.com

**Keywords:** artificial intelligence, triage, dermatology, value-based care

## Abstract

**Background/Objectives:** Demand for dermatologic services exceeds specialist capacity, with average wait times of 26–50 days in the United States. Current triage methods rely on subjective judgment or disease-specific indices that do not generalize across diagnoses or translate to operational decisions. We developed and validated the Dermatologic Complexity Score (DCS), a standardized instrument to guide case prioritization across dermatology care settings and evaluate DCS as a workload-reduction filter, enabling safe delegation of approximately half of routine teledermatology cases (DCS ≤ 40) away from specialist review. **Methods:** We conducted a prospective validation study of the DCS using 100 consecutive teledermatology cases spanning 30 common conditions. The DCS decomposes complexity into five domains (Diagnostic, Treatment, Risk, Patient Complexity, Monitoring) summed to a 0–100 total with prespecified bands: ≤40 (low) (41–70), (moderate) (71–89), (high), ≥90 (extreme). Five board-certified dermatologists and an automated module independently scored all cases. Two primary care physicians completed all ≤40 cases to assess feasibility. Primary outcomes were interrater reliability using ICC (2,1) and agreement with automation. Secondary outcomes included time-to-decision, referral rates, and primary care feasibility. **Results:** Mean patient age was 46.2 years; 47% of cases scored ≤40, 33% scored 41–70, 18% scored 71–89, and 2% scored ≥90. Interrater reliability was excellent (ICC (1,2)) = 0.979; 95% CI 0.974–0.983), with near-perfect agreement between automated and mean dermatologist scores (r = 0.998). Time-to-decision increased monotonically across DCS bands from 2.11 min (≤40) to 5 (90) min (≥90) (p = 1.36 × 10^−14^). Referral rates were 0% for ≤40, 3% for 41–70, 27.8% for 71–89, and 100% for ≥90 cases. DCS strongly predicted referral decisions (AUC = 0.919). Primary care physicians successfully managed all ≤40 cases but required 6–8 additional minutes per case compared to dermatologists. **Conclusions:** The DCS demonstrates excellent reliability and strong construct validity, mapping systematically to clinically relevant outcomes, including decision time and referral patterns. The instrument enables standardized, reproducible triage decisions that can optimize resource allocation across teledermatology, clinic, procedural, and inpatient settings. Implementation could improve access to dermatologic care by supporting appropriate delegation of low-complexity cases to primary care while ensuring timely specialist evaluation for high-complexity conditions.

## 1. Introduction

Demand for dermatologic services routinely exceeds specialist capacity, producing prolonged waits and suboptimal allocation of in-person visits [1,2,3]. This fundamental mismatch between healthcare needs and available resources has reached crisis proportions across healthcare systems globally, with profound implications for patient outcomes, healthcare costs, and system efficiency. Average wait times for dermatology appointments in the United States range from 26 to 50 days, with some areas experiencing delays exceeding 8 months [4,5,6]. These delays are not merely inconveniences; they represent a systematic barrier to timely diagnosis and treatment that can result in disease progression, worsened clinical outcomes, and increased healthcare utilization through emergency department visits and urgent care consultations.

The shortage of dermatologic services is particularly pronounced in underserved populations, where approximately 3.4 dermatologists serve per 100,000 people, well below adequate care thresholds [7]. This disparity creates a two-tiered system where geographic location, socioeconomic status, and insurance coverage become determinants of access to specialist care. Rural populations face even greater challenges, with many counties having no practicing dermatologists, forcing patients to travel hundreds of miles for consultation. The downstream consequences of delayed dermatologic care extend beyond individual patient impacts to broader public health concerns, including delayed melanoma diagnosis, progression of inflammatory skin diseases, and reduced quality of life for millions of patients with chronic dermatologic conditions.

Although asynchronous (“store-and-forward”) teledermatology expands access [8,9,10,11], reducing wait times and improving care delivery in resource-poor settings [12,13], the fundamental challenge of effective triage remains unresolved across all care modalities. Whether in traditional face-to-face clinics, synchronous video consultations, or asynchronous platforms, the decision-making process for resource allocation lacks systematic frameworks. Triage across all care settings, including clinic, telemedicine, procedural, and inpatient consults, often relies on subjective judgment [14,15] that varies significantly between providers and institutions. This variability creates inefficiencies, inequitable access patterns, and suboptimal resource utilization.

While asynchronous teledermatology expands access and expedites triage, it has important limitations: image quality and lighting variability can impair morphologic assessment; history elements may be incomplete; urgent lesions still require timely in-person confirmation; and workflow differences between sites can introduce spectrum and verification biases. Moreover, teledermatology does not itself standardize clinical complexity, leaving escalation thresholds and resource prioritization to local heuristics. These constraints underscore the need for a transparent, generalizable complexity instrument to guide triage across settings.

DermFlow (Delaware, USA) is an asynchronous telehealth platform that integrates automated quality filtering, complexity assessment, and case routing. When patients submit cases, the system first checks image quality and intake completeness, prompting resubmission if needed. It then calculates complexity scores and routes cases to appropriate providers based on thresholds.

Existing disease-specific indices, while valuable for research and monitoring individual conditions, do not generalize across diagnoses or map cleanly to operational decisions [16,17]. The Psoriasis Area and Severity Index (PASI), Dermatology Life Quality Index (DLQI), and similar instruments provide excellent disease-specific assessments but fail to create a universal framework for complexity assessment that can inform triage decisions across the full spectrum of dermatologic presentations. These tools typically focus on single conditions and do not account for patient-level factors, treatment complexity, or system-level resource requirements that drive operational decision-making.

Our objective was to validate the DCS for adult teledermatology cases that pass automated quality checks, thereby enabling more efficient utilization of specialist resources [18,19,20]. The Dermatologic Complexity Score (DCS) was developed to meet this need through a systematic, evidence-based approach to complexity measurement. Here, we validate the Dermatologic Complexity Score (DCS) as a standardized instrument for case prioritization, including its role as a triage filter to reduce teledermatologist workload by routing low-complexity cases (≤40) to non-specialist pathways.

## 2. Methods

**Figure 1:**
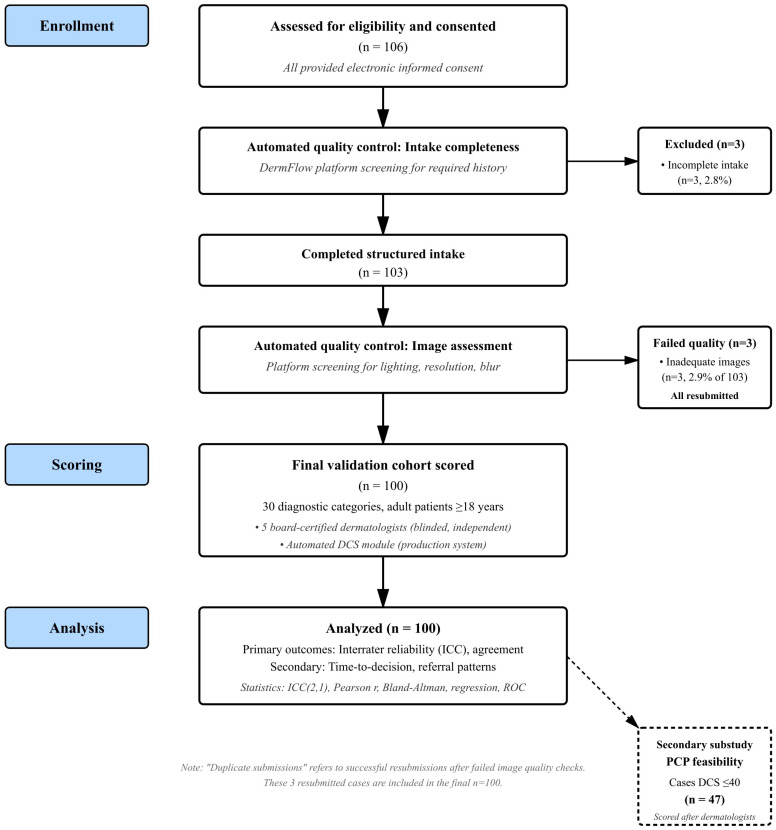
Participant flow diagram showing screening, enrollment, scoring, and outcomes. The PCP substudy represents a secondary feasibility analysis performed after DCS scoring was complete for low-complexity cases.

We conducted a prospective, observational validation study of the DCS embedded within DermFlow (Delaware, USA, https://dermflow.io, accessed on 2 March 2025), an asynchronous artificial intelligence-enabled teledermatology application that collects comprehensive targeted histories prior to physician review. The selection of teledermatology as the initial validation environment was deliberate and strategic, representing a controlled setting where standardized data collection, blinded review, and objective outcome measurement could be optimized. Teledermatology platforms provide natural laboratories for complexity assessment validation because they capture structured clinical data, standardize the review process, and generate measurable outcomes, including time-to-decision and referral patterns. A schematic of the workflow is provided in Figure 1.

The Indiana University IRB approved the protocol (IRB #28224) following a comprehensive review of study procedures, data security measures, and patient privacy protections. Adult participants provided electronic informed consent through a secure process that detailed study objectives, data usage, and privacy safeguards. The consent process included specific authorization for using de-identified clinical data and photographs for research purposes, with clear statements regarding data retention and potential future use in algorithm development and validation studies.

From March through May 2025, we enrolled 100 consecutive adult patients whose submissions passed automated quality screening and who provided electronic informed consent, providing a sample size adequate for reliability assessment while remaining manageable for comprehensive expert review. The sample size of 100 cases was selected to provide adequate precision for ICC estimation while remaining feasible for comprehensive expert review. For ICC reliability studies, a sample size of 100 with 5 raters provides ICC confidence interval widths of approximately 0.02–0.03 for true ICC values above 0.90, meeting conventional standards for reliability estimation. This sample size also enables detection of moderate correlations (r ≥ 0.30) between DCS and continuous outcomes with 80% power at α = 0.05. While larger samples would improve the precision of effect estimates, the current sample provides sufficient evidence for the initial validation of a novel instrument across diverse diagnostic categories. The sampling was truly consecutive: all adult submissions during the enrollment window were screened in the order received, and cases meeting inclusion criteria were enrolled sequentially until reaching our target sample size. We did not pre-select cases by diagnosis, complexity level, or any other clinical characteristic.

Inclusion criteria required complete structured intake data and adjudicable photographic images of sufficient quality for diagnostic assessment. Cases were excluded if intake data were incomplete, if photographs were of insufficient quality for clinical decision-making, or if they represented duplicate submissions from the same patient for the same condition. Eligibility included adults ≥18 years (IRB #28224); pediatric patients were excluded. The decision to exclude pediatric patients (under 18 years) was made for several methodological reasons. First, dermatologic complexity in pediatric populations requires distinct scoring considerations, including developmental factors, parent–child communication dynamics, and age-specific treatment constraints that warrant separate validation. Second, the DermFlow platform was designed and approved for adult populations, and extending validation to pediatrics would require separate IRB approval and parental consent procedures, which the current IRB was not designed for.

Each case contained a median of three photographs (IQR 2–4), reflecting typical patient submission patterns and providing multiple perspectives and anatomical views as clinically indicated. Consecutive submissions during the enrollment window were screened. Of 106 total screened submissions, all of whom signed consent, 3 (2.8%) were excluded for incomplete structured intake, leaving 103 that completed intake. Three cases were excluded for inadequate/blurred images (*n* = 3) and duplicate submissions (*n* = 3). These duplicate submissions and inadequate images were the same cases, as the platform requested a resubmission when the image quality check failed, leaving 100 total analyzed cases. All exclusions were automatically identified by the DermFlow platform’s quality control module prior to entry into the study queue. The platform employs automated quality checks for image resolution, lighting adequacy, and validation of the dermatologic nature of the image(s), prompting users to resubmit when standards are not met. This automatic filtering represents core functionality of the production system and is central to its real-world clinical utility. For this validation study, cases that failed automated quality checks and required resubmission were counted as exclusions. The three duplicate submissions were from the same patients whose initial images were flagged as inadequate and subsequently resubmitted with improved quality.

The DCS comprises five carefully designed domains that collectively capture the multidimensional nature of dermatologic case complexity. The Diagnostic domain (0–25 points) assesses the degree of diagnostic uncertainty, ranging from pathognomonic presentations that can be confidently diagnosed from clinical appearance alone to complex cases requiring histopathologic confirmation or subspecialty consultation. Scoring criteria include the need for differential diagnosis consideration, requirement for additional testing (dermoscopy, biopsy, laboratory studies), and likelihood of diagnostic accuracy based on clinical presentation alone.

The Treatment domain (0–25 points) evaluates therapeutic intervention complexity, spanning simple topical therapies manageable with basic patient education to complex systemic treatments requiring specialized monitoring, drug interactions assessment, and coordinated subspecialty care. Scoring considers the number of therapeutic agents, potential for significant side effects, laboratory monitoring requirements, and complexity of patient education protocols. The Risk domain (0–25 points) captures patient-specific and condition-specific factors elevating adverse outcome potential, including malignancy risk, rapid progression potential, immunosuppression, pregnancy status, and social or environmental factors affecting treatment adherence, while also considering consequences of delayed or inappropriate treatment ranging from cosmetic concerns to life-threatening complications.

The Patient Complexity domain (0–15 points) addresses patient-specific factors influencing care delivery beyond medical risk, including health literacy levels, language barriers, psychiatric comorbidities affecting adherence, complex social situations, compliance history, multiple active comorbidities requiring care coordination, polypharmacy concerns, and patient preference factors complicating standard approaches. The Monitoring domain (0–10 points) evaluates follow-up care intensity and duration, ranging from single-visit resolutions requiring no ongoing monitoring to complex conditions necessitating frequent reassessment, laboratory monitoring, and long-term specialist oversight, considering both frequency and complexity of required assessments, specialized monitoring procedures, and potential for treatment modifications based on response or side effects.

Each domain includes detailed anchoring criteria that specify the clinical factors and thresholds corresponding to different score ranges within that domain. These anchors were developed through extensive clinical consensus processes involving board-certified dermatologists with expertise in various subspecialties and care settings. The anchoring criteria are sufficiently detailed to enable consistent application by different raters while maintaining clinical relevance and face validity across diverse case presentations.

Five board-certified dermatologists with varying subspecialty expertise and practice settings independently reviewed every case within the DermFlow platform. Reviewers were blinded to one another’s assessments and to the automated DCS to ensure independent judgment. Each dermatologist recorded the five domain subscores based on the standardized anchoring criteria, calculated the total DCS (sum of all domain scores), measured time-to-decision (minutes elapsed from initial case opening to final disposition entry), and determined case disposition (managed within the teledermatology platform versus referred for in-person evaluation). The time-to-decision measurement captured actual clinical decision-making time rather than total case review time, providing an objective measure of cognitive complexity and effort required for case resolution.

The index test was DermFlow’s production DCS module, which generated automated complexity scores at the time of case opening according to the prespecified domain anchoring criteria. The automated scoring algorithm processed structured intake data and applied rule-based logic to assign domain scores without human intervention. Importantly, no algorithm retraining, parameter tuning, or optimization occurred during the validation study period, ensuring that the automated scores reflected the performance of the production system rather than an optimized research implementation.

To assess the feasibility and safety of complexity-based care delegation to primary care providers, two experienced primary care physicians independently reviewed all cases with DCS ≤ 40. These PCPs used the DermFlow platform’s automated DCS scoring to identify eligible cases but did not perform an independent complexity rating. Instead, they focused on case management decisions, recording time-to-decision and treatment recommendations. Their dispositions were compared with dermatologist recommendations for the same cases to assess concordance and identify potential safety concerns with primary care delegation of low-complexity cases.

### Statistical Methods Used

We evaluated interrater reliability using a two-way random-effects, absolute-agreement intraclass correlation coefficient, ICC (2,1), with 95% confidence intervals via nonparametric bootstrap. Agreement between automated and mean dermatologist scores was assessed with Pearson correlation and Bland–Altman analysis (bias and limits of agreement). Construct validity was examined by modeling decision time as a function of DCS using ordinary least squares regression and one-way ANOVA across prespecified complexity bands. Triage validity was evaluated using logistic regression of in-person referral on DCS and by computing the area under the receiver operating characteristic curve (AUC). Two-sided alpha = 0.05 defined statistical significance. Analyses were performed in Python v.3.11 with statsmodels and scikit-learn (or equivalent packages).

## 3. Results

**Figure 2:**
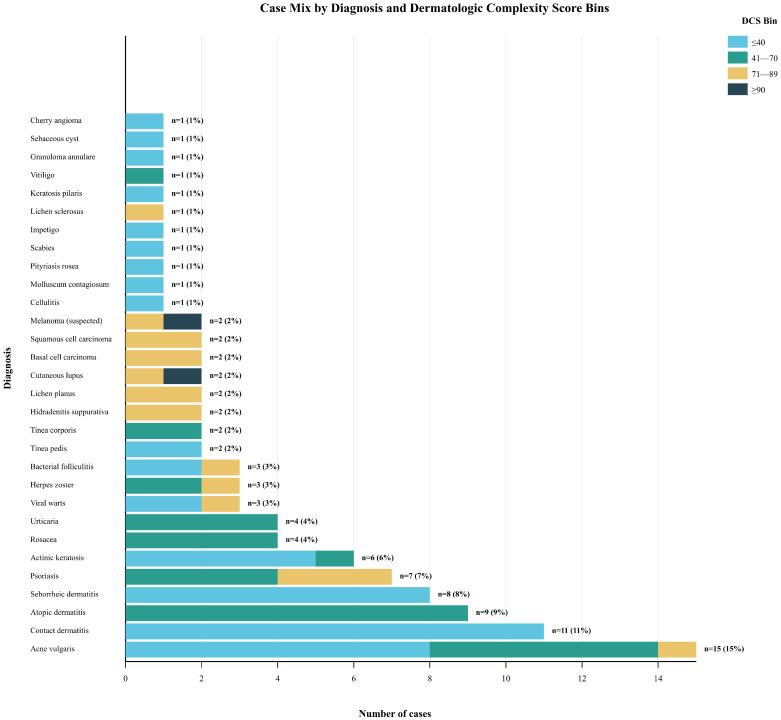
Case Mix by Diagnosis and Dermatologic Complexity Score Bins.

During the study period, 106 submissions were received; 5.7% were excluded, yielding 100 consecutive, unique teledermatology cases for analysis, representing a comprehensive sample of common outpatient dermatologic presentations. The consecutive sampling yielded a diagnostically diverse cohort reflecting a typical teledermatology case mix. The 100 cases encompassed 30 distinct diagnostic categories, with a distribution of cases that resembles the diagnostic frequency patterns observed in both primary care and dermatology specialty settings (Figure 2).

The cohort demographics reflected typical teledermatology utilization patterns with a mean age of 47.8 years (SD 17.0; median 46; IQR 33–60) and slight female predominance (56%). The Fitzpatrick phototype distribution demonstrated reasonable diversity: Type I 15%, Type II 24%, Type III 27%, Type IV 20%, Type V 9%, and Type VI 5%. Ten percent of patients were immunocompromised due to medications or underlying conditions, and 3% were pregnant, representing important high-risk subpopulations requiring specialized consideration. Comorbidity burden averaged 0.78 conditions per patient (SD 0.92; median 1; IQR 0–1), reflecting the relatively healthy population typically accessing elective dermatologic care through teledermatology platforms (Table 1) [21].

The case mix nearly successfully captured the intended breadth of common dermatologic conditions, aside from a slight underrepresentation of the 18–29 age group. The underrepresentation of patients under 30 years (9% of the cohort) reflects a limitation of the consecutive sampling approach during the enrollment window. The lower proportion of acne vulgaris cases (typically more common in younger populations) suggests potential sampling bias, maybe related to time-of-day submission patterns, or the fact that DermFlow is primarily currently serving Medicare and Medicaid populations. As patients under 26 may be on their parents’ insurance, the commercial payer mix may currently be unintentionally excluded. To improve representativeness, we recommend that future multi-site validation studies stratify enrollment by time of day and actively recruit during evening and weekend hours to better capture younger adult utilization patterns. This limitation should be considered when generalizing DCS performance to younger populations, though the scoring system’s domain structure remains applicable across age groups.

Analysis of median DCS by diagnostic category revealed clinically intuitive patterns that supported the face validity of the complexity measurement approach. Conditions traditionally considered straightforward and suitable for primary care management clustered in lower complexity ranges: acne vulgaris (median DCS 28), contact dermatitis (median DCS 32), and seborrheic dermatitis (median DCS 35). Moderate complexity conditions showed intermediate scores: atopic dermatitis (median DCS 52), psoriasis (Median DCS 58), and rosacea (median DCS 45). High-risk conditions consistently scored in elevated ranges: cutaneous lupus erythematosus (median DCS 78), suspected melanoma (median DCS 85), and hidradenitis suppurativa (median DCS 72). Notably, none of the high-risk entities (cutaneous lupus or suspected malignancies) fell into the ≤40 complexity band, supporting the safety and clinical validity of the proposed triage thresholds (Table 2). Operationally, this implies a potential ~47% reduction in teledermatologist case load if DCS ≤40 cases are routed to supported non-specialist pathways.

The distribution of total DCS values followed a right-skewed pattern typical of complexity measures in healthcare settings, where most cases represent routine presentations with a minority requiring intensive resources. Forty-seven cases (47%) scored ≤40, representing the population potentially suitable for primary care management with appropriate support systems. Thirty-three cases (33%) fell into the moderate complexity range (41–70), representing cases that could be managed flexibly across different care modalities based on local resources and patient factors. Eighteen cases (18%) were classified as high complexity (71–89), requiring specialist oversight and potentially expedited access. Only two cases (2%) reached the extreme complexity threshold (≥90), representing urgent specialist-required presentations. This distribution pattern suggests that approximately 80% of routine dermatologic presentations score ≤ 70, with roughly half being potentially appropriate for alternative care delivery models (Table 2).

Interrater reliability analysis demonstrated exceptional consistency across the five dermatologists and the automated DCS module. The intraclass correlation coefficient ICC(2,1) was 0.979, with a narrow bootstrap 95% confidence interval of 0.974–0.983, indicating near-perfect agreement among raters. This level of reliability substantially exceeds the conventional thresholds for excellent agreement (ICC ≥ 0.75) and approaches the theoretical maximum for human rating tasks. The consistency of agreement across both human raters and the automated system demonstrates that the explicit domain anchoring criteria are sufficiently clear and comprehensive to enable reproducible complexity assessment across different evaluators and implementation modalities (Table 3).

Agreement between the automated DermFlow scoring system and mean dermatologist scores approached mathematical identity, with a Pearson correlation coefficient of r = 0.998. Bland–Altman analysis revealed minimal systematic bias, with the automated system showing a small positive bias of +0.72 points (95% limits of agreement −2.58 to +4.03 points). This near-perfect agreement indicates that the algorithmic implementation accurately captures the clinical reasoning embedded in the domain anchoring criteria and can serve as a reliable proxy for expert human assessment. The minimal bias and narrow limits of agreement support the use of automated scoring for real-time clinical decision support and large-scale implementation across healthcare systems (Table 3).

Analysis of practical triage agreement examined clinician consensus on operationally relevant complexity categories: clearly primary care appropriate (≤20), borderline cases requiring judgment (21–40), and specialist-managed cases (≥41). Overall agreement was substantial (Fleiss’ κ = 0.79), with notably higher concordance at the extremes of the complexity spectrum. Agreement was highest for clearly low-complexity cases (≤20: 97% agreement) and clearly high-complexity cases (≥41: 95% agreement), with somewhat lower but still substantial agreement in the borderline range (21–40: 82% agreement). This pattern supports the clinical utility of the DCS for operational decision-making, as the highest reliability occurs precisely where triage decisions have the greatest impact on patient safety and resource allocation.

The relationship between complexity scores and operational outcomes provided strong evidence for construct validity and clinical utility. Dermatologist time-to-decision increased in a clear, stepwise fashion across complexity bands, demonstrating that the DCS captures objective measures of clinical effort and cognitive complexity. Mean decision times were 2.11 min (SD 0.66; 95% CI 1.92–2.30) for low complexity cases (≤40) 2.72 min (SD 0.77; 95% CI 2.46–2.98) for moderate complexity (41–70) 3.72 min (SD 0.67; 95% CI 3.46–3.99) for high complexity (71–89), and 5.90 min (SD 0.14) for extreme complexity cases (≥90). The omnibus ANOVA demonstrated highly significant differences between groups (F = 32.52, *p* = 1.36 × 10^−14^), confirming that complexity scores correspond to measurable differences in clinical effort and resource utilization (Table 4).

Continuous modeling of the time-complexity relationship revealed that each 1-point increase in DCS was associated with an additional 0.0276 min of decision time (95% CI 0.0200–0.0351; *p* = 7.37 × 10^−13^), with the complexity score explaining 43.5% of the variance in decision time (R^2^ = 0.435). This strong linear relationship supports the use of DCS for operational planning and resource allocation, as complexity scores provide predictive information about the time and effort required for case resolution across the full range of dermatologic presentations (Table 4).

Referral patterns across complexity bands demonstrated the clinical validity of the scoring system for triage decision-making. Referral rates increased dramatically with complexity scores: 0% for cases ≤40, 3% for moderate complexity cases (41–70), 27.8% for high complexity cases (71–89), and 100% for extreme complexity cases (≥90). Logistic regression modeling confirmed that DCS strongly predicted referral decisions, with each 10-point increase in complexity score associated with an odds ratio of 3.35 for in-person referral (95% CI 1.50–7.47; *p* = 0.0031). The discriminative ability of the DCS for predicting referral was excellent, with an area under the receiver operating characteristic curve (AUC) of 0.919, indicating that complexity scores can reliably distinguish between cases appropriate for different care modalities (Table 4).

The primary care feasibility assessment provided important evidence regarding the safety and practicality of complexity-based care delegation. Primary care physicians successfully completed all cases with DCS ≤ 40, with clinical dispositions that were concordant with dermatologist management recommendations, suggesting that appropriately selected low-complexity cases can be safely managed in primary care settings. However, PCPs required significantly more time than dermatologists for case resolution. The mean additional time was 6.48 min (SD 0.82; 95% CI 6.13–6.83; *p* = 5.49 × 10^−20^) for very low complexity cases (1–20) and 7.93 min (SD 1.11; 95% CI 7.50–8.35; *p* = 1.67 × 10^−23^) for borderline cases (21–40, Table 5).

## 4. Discussion

Beyond the immediate teledermatology validation context, the DCS framework has broad applicability across the full spectrum of dermatologic care delivery models. In-person specialty clinics can implement DCS at the time of scheduling to optimize session templates and appointment duration allocation, ensuring that complex cases receive adequate time while maximizing access for routine presentations. The scoring system can inform decisions about same-day urgent slots, provider assignment based on expertise levels, and coordination with ancillary services such as pathology or photography.

Synchronous teledermatology platforms can utilize DCS for real-time prioritization, ensuring that high-complexity cases receive same-day video review while routing appropriate cases through asynchronous workflows. The automated scoring capability enables immediate complexity assessment at the point of case submission, facilitating dynamic queue management and resource allocation without requiring preliminary physician review. This capability is particularly valuable for large-scale teledermatology programs managing hundreds of cases daily across multiple providers and geographic regions.

The time differential observed between specialists and primary care providers reflects predictable differences in clinical experience and pattern recognition capabilities. This gap can be addressed through targeted education programs, clinical decision support tools, standardized treatment protocols, and readily accessible specialist consultation. By enabling appropriate matching of clinical resources to patient needs, complexity-based triage can reduce both overutilization of specialist resources for routine cases and underutilization of specialists for complex presentations, potentially improving access and efficiency across healthcare systems.

The potential for algorithmic bias and health equity concerns requires ongoing attention and monitoring in DCS implementations. While the current validation cohort demonstrated reasonable demographic diversity, broader implementation across more diverse populations will require careful assessment of scoring accuracy and fairness across different patient groups. Particular attention must be paid to ensuring that complexity assessments do not inadvertently reflect or amplify existing healthcare disparities, and that access to high-quality care remains equitable across all patient populations, regardless of complexity scores.

Human factors research focusing on clinician acceptance, workflow integration, and decision-making processes will optimize practical implementation [22], addressing both technical usability and organizational change management challenges.

### Limitations

Findings derive from an asynchronous teledermatology context and a modest single-platform sample, which may limit generalizability to synchronous telehealth or in-person settings. The exclusion of cases with incomplete histories or poor-quality images represents both a study limitation and a critical feature of real-world implementation. The DermFlow platform addresses this challenge through automated quality control: incomplete intake triggers specific follow-up questions before physician review, and inadequate images prompt immediate resubmission with guidance on lighting and positioning. This automated filtering occurred prior to any cases entering our validation cohort. While this ensures high-quality data for complexity assessment, it represents a best-case scenario. In practice, healthcare systems implementing DCS without robust automated quality control may encounter cases where complexity cannot be reliably scored, limiting clinical utility. The current validation, therefore, demonstrates DCS performance when supported by adequate data infrastructure, not its performance with unstructured or incomplete clinical data. Future research should evaluate DCS accuracy when applied to suboptimal submissions to determine minimum data quality thresholds for safe complexity assessment.

Although we demonstrated strong reliability and validity signals, outcomes (e.g., diagnostic accuracy, treatment response) were not longitudinally adjudicated. Band thresholds were prespecified for operational feasibility and observed to perform well in this cohort, but will require prospective tuning across diverse sites and patient populations, including equity analyses across skin phototypes. Cohort composition may limit representativeness: pediatric patients were excluded by design, and the intake source during the study window yielded a low proportion of patients <30 years. Limited size and adult-only samples may, therefore, limit external validity. These factors may under-represent common conditions in younger populations and should be addressed in future all-comer, multi-site validations.

## 5. Conclusions

This validation demonstrates the utility of the DCS as a potential tool for complexity assessment in adult teledermatology with automated quality controls. The excellent interrater reliability (ICC = 0.979) and automation agreement (r = 0.998) demonstrate that standardized complexity measurement may be possible in dermatologic presentations, with strong relationships to objective outcomes suggesting potential real-world applicability. Operationally, nearly half of the teledermatology cases can be identified as low-complexity (DCS ≤ 40) and potentially delegated to primary care. While primary care physicians managed these cases safely, the 6–8-min time differential raises important questions about workflow efficiency and economic sustainability that must be resolved before implementation. The conclusions demonstrated in this study apply specifically to cases pre-filtered by automated quality controls. Systems lacking such infrastructure may encounter unsuitable cases. The DCS provides an explainable framework for complexity assessment that, with appropriate governance measures including prospective monitoring and periodic recalibration [23], could optimize dermatologic care delivery.

## Figures and Tables

**Table 1:** Demographics (Expanded).

Characteristic	Value
*N*	100
Age, mean (SD)	47.8 (15.0)
Age, median (IQR)	50 (37–57)
Sex: Female *n* (%)	56 (56.0%)
Sex: Male *n* (%)	44 (44.0%)
Fitzpatrick I *n* (%)	13 (13.0%)
Fitzpatrick II *n* (%)	29 (29.0%)
Fitzpatrick III *n* (%)	26 (26.0%)
Fitzpatrick IV *n* (%)	18 (18.0%)
Fitzpatrick V *n* (%)	8 (8.0%)
Fitzpatrick VI *n* (%)	6 (6.0%)
Age 18–29 *n* (%)	9 (9.0%)
Age 30–44 *n* (%)	37 (37.0%)
Age 45–64 *n* (%)	42 (42.0%)
Age 65+ *n* (%)	12 (12.0%)
Immunocompromised *n* (%)	7 (7.0%)
Pregnant *n* (%)	3 (3.0%)
Comorbidities, mean (SD)	0.78 (0.92)
Comorbidities, median (IQR)	1 (0–1)

**Table 2:** Scores, Time, and Referrals by Band.

Band	*N*	Referred *n* (%)	Derm Time Mean (SD), min	Derm Time 95% CI, min	Derm Time Median (IQR), min	Score Median (IQR)
≤40	47	0 (0.0%)	2.11 (0.66)	1.92–2.30	2.21 (1.64–2.62)	22 (12–32)
41–70	33	1 (3.0%)	2.72 (0.77)	2.46–2.98	2.77 (2.31–3.23)	58 (47–62)
71–89	18	5 (27.8%)	3.70 (0.93)	3.27–4.13	3.79 (3.21–4.23)	76 (74–82)
≥90	2	2 (100.0%)	5.90 (0.14)	5.70–6.10	5.90 (5.85–5.95)	93 (92–94)

**Table 3:** Reliability and Agreement.

Metric	Estimate	95% CI
ICC(2,1), 5 derms + DermFlow	0.979	0.974–0.983
Pearson r, DermFlow vs. mean derm	0.998	
Fleiss κ, triage categories (all)	0.793	
Fleiss κ, ≤20	−0.029	
Fleiss κ, 21–40	0.098	
Fleiss κ, ≥41	0.083	
Bland–Altman bias	0.72	NA
Bland–Altman limits of agreement	−2.58 to 4.03	NA

**Table 4:** Modeling (Time and Referral).

Analysis	Estimate
Linear: Time ~ Score (per point)	β = 0.028 (95% CI 0.020 to 0.035), *p* = 0.0000, R^2^ = 0.435
ANOVA: Time by Band	F = 32.52, *p* = 1.3649 × 10^−14^
Logistic: Referral ~ Score (per 10 points)	OR = 3.35 (95% CI 1.50–7.47), *p* = 3.0986 × 10^−3^
Discrimination: AUC (Score → Referral)	AUC = 0.919

**Table 5:** PCP vs. Dermatologist Time (≤40).

Bucket	*N*	Mean Δ (SD), min	95% CI	*t*-Test vs. 0 *p*
1–20	21	6.48 (0.82)	6.13–6.83	5.485 × 10^−20^
21–40	26	7.93 (1.11)	7.50–8.35	1.6738 × 10^−23^

## Data Availability

The data presented in this study are available on request from the corresponding author due to academic institutional restrictions.
